# Probiotics Modulate Intestinal Expression of Nuclear Receptor and Provide Counter-Regulatory Signals to Inflammation-Driven Adipose Tissue Activation

**DOI:** 10.1371/journal.pone.0022978

**Published:** 2011-07-29

**Authors:** Andrea Mencarelli, Eleonora Distrutti, Barbara Renga, Claudio D'Amore, Sabrina Cipriani, Giuseppe Palladino, Annibale Donini, Patrizia Ricci, Stefano Fiorucci

**Affiliations:** 1 Dipartimento di Medicina Clinica e Sperimentale, University of Perugia, Facoltà di Medicina e Chirurgia, Via Gerardo Dottori n° 1 S. Andrea delle Fratte, Perugia, Italy; 2 Azienda Ospedaliera di Perugia, Ospedale Santa Maria della Misericordia, S. Andrea delle Fratte, Perugia, Italy; 3 Dipartimento di Scienze Chirurgiche, Radiologiche e Odontostomatologiche, Nuova Facoltà di Medicina e Chirurgia Sant' Andrea delle Fratte, Perugia, Italy; Università degli Studi di Milano, Italy

## Abstract

**Background:**

Adipocytes from mesenteric white adipose tissue amplify the inflammatory response and participate in inflammation-driven immune dysfunction in Crohn's disease by releasing proinflammatory mediators. Peroxisome proliferator-activated receptors (PPAR)-α and -γ, pregnane x receptor (PXR), farnesoid x receptor (FXR) and liver x-receptor (LXR) are ligand-activated nuclear receptor that provide counter-regulatory signals to dysregulated immunity and modulates adipose tissue.

**Aims:**

To investigate the expression and function of nuclear receptors in intestinal and adipose tissues in a rodent model of colitis and mesenteric fat from Crohn's patients and to investigate their modulation by probiotics.

**Methods:**

Colitis was induced by TNBS administration. Mice were administered vehicle or VSL#3, daily for 10 days. Abdominal fat explants obtained at surgery from five Crohn's disease patients and five patients with colon cancer were cultured with VSL#3 medium.

**Results:**

Probiotic administration attenuated development of signs and symptoms of colitis, reduced colonic expression of TNFα, IL-6 and IFNγ and reserved colonic downregulation of PPARγ, PXR and FXR caused by TNBS. Mesenteric fat depots isolated from TNBS-treated animals had increased expression of inflammatory mediators along with PPARγ, FXR, leptin and adiponectin. These changes were prevented by VSL#3. Creeping fat and mesenteric adipose tissue from Crohn's patients showed a differential expression of PPARγ and FXR with both tissue expressing high levels of leptin. Exposure of these tissues to VSL#3 medium abrogates leptin release.

**Conclusions:**

Mesenteric adipose tissue from rodent colitis and Crohn's disease is metabolically active and shows inflammation-driven regulation of PPARγ, FXR and leptin. Probiotics correct the inflammation-driven metabolic dysfunction.

## Introduction

Crohn's disease is a chronic and progressive inflammatory disorder of gastrointestinal tract. Transmural inflammation is the histological hallmark of Crohn's disease with inflammation extending beyond the intestinal wall. An involvement of mesenteric adipose tissue (MAT) is increasingly thought to provide a mechanistic contribution to disease progression. This contention is supported by the fact that at the onset of disease, although patients show a weight loss, [1] a specific hypertrophy of MAT is frequently identified [1]–[3]. Fat wrapping extending from the mesenteric attachment and partially covering the intestinal circumference, is common in both the small and large intestine and is also considered a hallmark of Crohn's disease [1]. This ectopic tissue is referred to as “creeping fat” and is encroached at the antimesenteric surface of the bowel. Surgeons are familiar with creeping fat and use it as an anatomical marker to delineate the extent of active disease [4]. Adipose tissue is increasingly identified as a major endocrine organ from which either metabolic and inflammatory signals propagate systemically, potentially modulating clinical features of Crohn's disease. In Crohn's disease patients creeping fat is infiltrated by activated macrophages and releases high amount of TNF-α and leptin, a proinflammatory adipokine, indicating that this tissue could play a mechanistic role in maintaining local and systemic inflammation [5], [6]. In chronic disorders such as diabetes and obesity MAT hypertrophy is governed by the activation of a family of nuclear receptors- peroxisome proliferator-activated receptor (PPAR) α and γ, farnesoid-x-receptor (FXR) and liver–x-receptor (LXR)- and adipokynes that are well identified targets for medical interventions. In contrast it is still unknown whether MAT could be modulated by pharmacological interventions in Crohn's disease [7]. The commensal gut microbiota has profound effects on the physiology of the host [8]. The intestinal microbiota may be exerting effects beyond the intestine and patients with chronic inflammatory disorders such as obesity and Type I diabetes display an altered gut flora that may have a pathogenetic readout on the phenotype of these disorders [8]–[10]. It is increasingly appreciated that circulating levels of xeno- and endo-biotics including bile acids, lipids and metabolism intermediates are regulated by gut microbiota [11]–[13]. Nutrients and metabolites are transported from the intestine to the liver via the afferent pathways, the i.e. the portal vein and the lymphatic system exerting a wide range of regulatory functions in abdominal tissues beyond the intestinal wall. Decoding the interactions between metabolic intermediates and endogenous receptors has lead to understanding that some of these metabolites act as ligands or activators for nuclear receptors, a large family of ligand activated regulatory factors that exert their homeostatic functions at the interface between nutrients metabolism and innate immunity. Thus, activation of PPAR-α and γ, FXR and LXR by lipid mediators, bile acids and oxysterols modulates lipid/cholesterol metabolism but also provides counter-regulatory signals for cells of innate immunity [14]-[18]. Probiotics which deliver some of the beneficial immunomodulatory effects of the commensal gut microbiota and induce immune homeostasis have been proposed as a suitable treatment for mild to moderate IBD. Probiotics intervention results in a site-specific reduction of inflammatory pathways with an increased expression of mediators involved in PPAR signalling, a pathway that is counter-regulatory for NF-κB [19]. The beneficial effects of probiotics extend outside the intestine and probiotics have been shown to exert a beneficial effects in obesity, NASH and diabetes despite the mechanisms mediating these effects have not been elucidated [20]. In this study we have investigated whether intestinal inflammation modulates the expression of nuclear receptors in the intestine and MAT in a rodent model of colitis and whether this pattern could be regulated by administration of probiotics. In addition, we have investigated whether this findings might have a translational relevance by examining the expression of nuclear receptors and adipokines in explants of creeping fat and MAT from Crohn's disease patients. Finally, by culturing creeping fat and MAT explants from Crohn's disease patients with conditioned medium from probiotics cultures we have provided evidence that probiotics modulate leptin release.

## Results

### Anti-Inflammatory effects of VSL#3 the TNBS model of colitis

**Figure 1:**
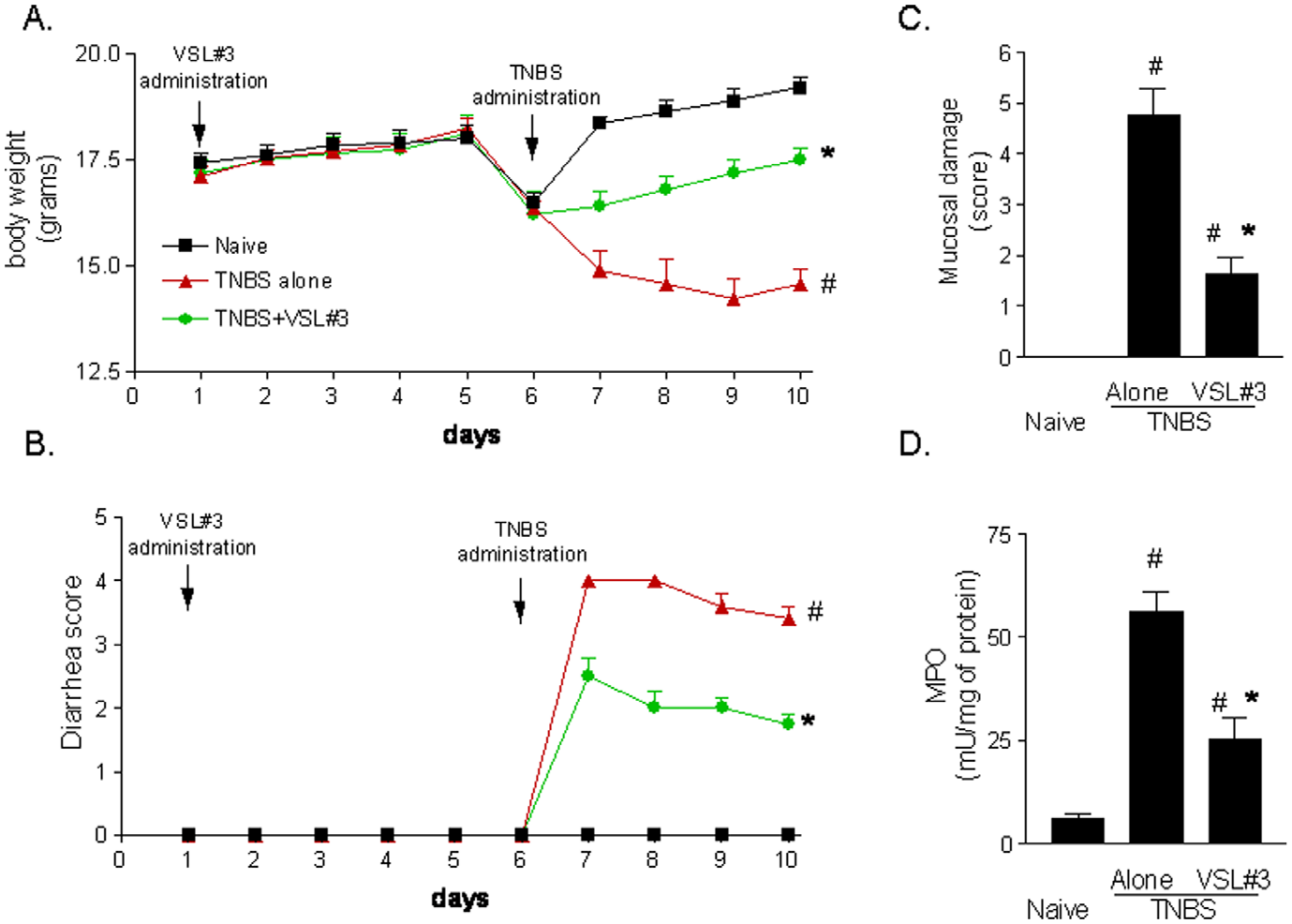
Anti-inflammatory activity of VSL#3 in TNBS colitis. Preteatment with VSL#3 (50×10^9^ colony-forming units (cfu)/kg/day) protects against the development of TNBS-induced colitis in mice. Colitis was induced by intrarectal instillation of 1.5 mg of TNBS per mouse. Mice were sacrificed 5 days after TNBS administration. (**A** and **B**) The severity of TNBS-induced inflammation (weight loss and fecal score) is reduced by VSL#3 administration. Data represent the mean ± SE of 8–10 mice per group. (#p<0.05 versus naïve; *p<0.05 versus TNBS). (**C** and **D**) VSL#3 reduces local signs of inflammation and inhibits the increase of macroscopic-score and neutrophil infiltration (MPO activity) induced by TNBS. Data represent the mean ± SE of 8–10 mice per group. (#p<0.05 versus naïve; *p<0.05 versus TNBS).

**Figure 2:**
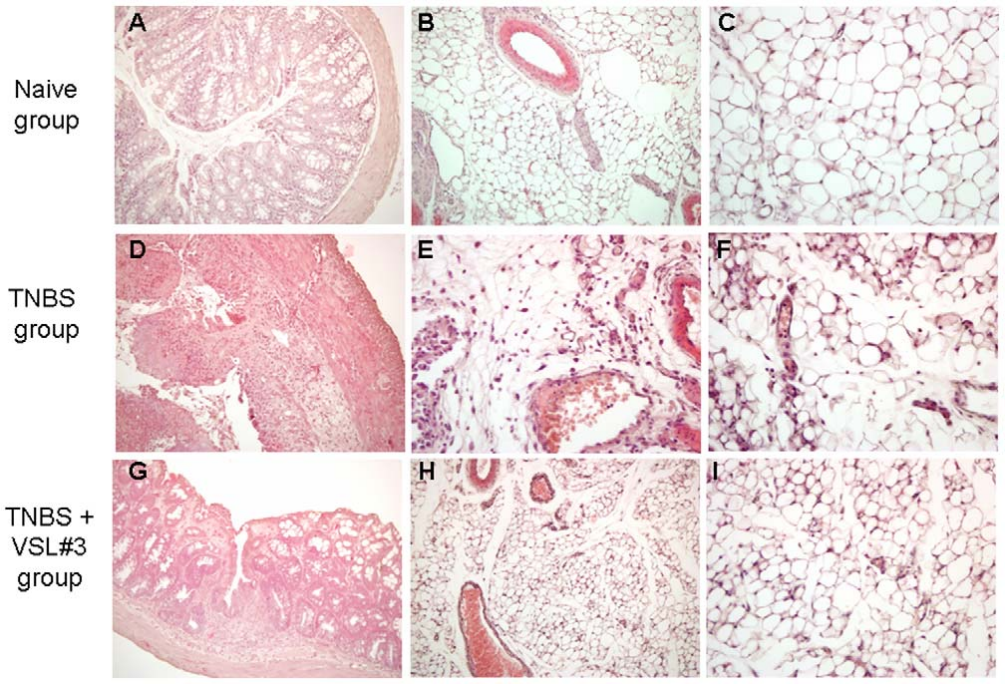
Histological analysis of colon and mesenteric adipose tissue of mice treated with TNBS alone or in combination with VSL#3. (**A, D and G**) Histopathology analysis of colon samples, original magnification 10×; H&E staining. (**A**) naïve mice; (**D**) TNBS administration causes colon wall thickening and massive inflammatory infiltration in the *lamina propria* and mucosal erosions*;* (**G**) VSL#3 attenuates colon thickening and inflammatory infiltration of the mucosa and submucosa. (**B,C,E,F,H and I**) Histologic analysis of mesenteric adipose tissue, H&E staining. (**B and C**) Naïve mice, original magnification 10× and 20×; (**E and F**) TNBS group mice, original magnification 10× and 20×; (**H and I**) VSL#3 treated mice, original magnification 10× and 20x.

**Figure 3:**
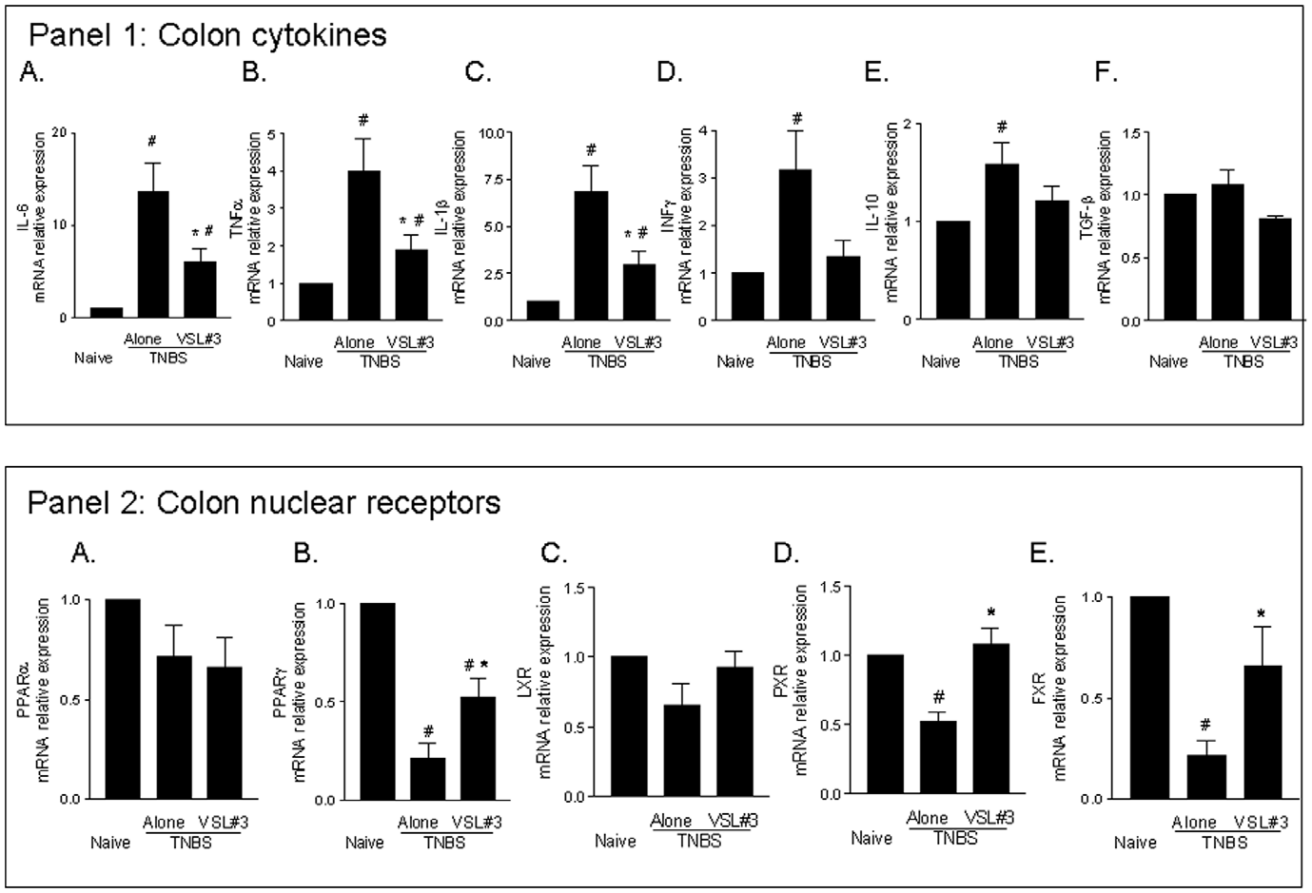
VSL#3 attenuates inflammatory changes in the colon and restores nuclear receptors expression in mice administered TNBS. (**Panel 1. A–F**) RT-PCR analysis of the expression of inflammatory cytokines (IL-6, TNFα, IL-1β, and INFγ) and anti-inflammatory cytokines (IL-10 and TGF-β) in colons obtained 5 days after TNBS. Data represent the mean ± SE of 5 mice per group. (#p<0.05 versus naïve; *p<0.05 versus TNBS). (**Panel 2** **A–E**) RT-PCR analysis of the expression of PPARα, PPARγ, FXR, LXR, PXR and CAR in colons removed 5 days after administration of TNBS alone or in combination with VSL#3. Data represent the mean ± SE of 5 mice per group. (#p<0.05 versus naïve; *p<0.05 versus TNBS).

Colon inflammation that develops in mice administered TNBS is thought to be a model of Th1-mediated disease with dense infiltrations of lymphocytes/macrophages in the *lamina propria* and thickening of the colon wall [18], [21]. The TNBS colitis is therefore characterized by typical sign and symptoms of colitis, including weight loss, diarrhea, macroscopic inflammation and prototypical histological and biochemical intestinal changes including increased levels of myeloperoxidase (MPO) activity, a biochemical marker of neutrophils infiltration. No changes in body weight were recorded in mice administered ethanol alone and followed for 7 days after TNBS administration (data not shown). In order to assess whether VSL#3 would exert immune-modulatory activity, mice administered TNBS were treated with VSL#3 (50×10^9^ colony-forming units (cfu)/day) for 5 days before induction of colitis. VSL#3 pretreatment effectively attenuated colitis development as measured by assessing local and systemic signs of inflammation. VSL#3 administration protected against the development of wasting disease measured by the weight loss and the diarrhea score (Figure 1A and 1B; n = 8–10, #p<0.05 versus naive group; *p<0.05 versus TNBS group) and reduced the macroscopic score of colitis (Figure 1 C; #p<0.05 versus control group; *p<0.05 versus TNBS group); moreover, it attenuated colon neutrophils infiltration, as measured by assessing the colonic activity of MPO (Figure 1 D #p<0.05 versus naive group; *p<0.05 versus TNBS group). Mortality was 10% per group and was related to TNBS inoculation. Indeed, mice deaths occurred mostly on day 7, i.e. one day after TNBS inoculation (data not shown). Figure 2 (panel A,D and G) illustrates a representative image of histopathological analysis of colons obtained from each treatment group. Compared with colons of naïve mice, colons obtained from mice administered with TNBS showed an extensive cellular infiltrate, submucosal edema and large areas of epithelial erosions. These changes were robustly attenuated by VSL#3 treatment. To gain insights on the immune-phenotype of colon lamina propria mononuclear cells (LPMC), the phenotype of LPMC cells isolated from animals administered TNBS alone or in combination with VSL#3 was compared to LPMC isolated from naïve mice. The immune-phenotypic characterization of these cells by CD3, CD19, CD14 and NK1.1 antibodies reveled that, in comparison to TNBS treated mice, VSL#3 co-administration caused a slight, thought significant, reduction in the percentage of CD14+ and CD3 +cells (see Figure S1). This effect associates with a robust attenuation of colonic expression of inflammatory and immune mediators including IL-6, TNFα, IL-1β and INFγ caused by TNBS (Figure 3 Panel 1A, B, C and D ; n = 5; #p<0.05 versus naïve; *p<0.05 versus TNBS), while failed to change the mRNA levels of IL-10 and TGF-β (Figure 3 Panel 1 E and F).

A growing body of evidence supports the notion that a mutual inhibition between pro-inflammatory mediators and nuclear receptors does exist in inflammatory bowel diseases (IBDs). [22], [23]. Consistent with this view, we found that acute TNBS-colitis causes a robust decrease in the expression of several nuclear receptor including PPARγ, PXR and FXR. These changes were antagonized by VSL#3 cotreatment (Figure 3, Panel 2; n = 5; #p<0.05 versus naïve; *p<0.05 versus TNBS). These changes in gene product expression are mirrored by changes at the protein levels, as demonstrated by the analysis of the colonic expression of FXR protein (see Figure S2).

### Effects of VSL#3 administration on the mesenteric adipose tissue in mouse colitis

**Figure 4:**
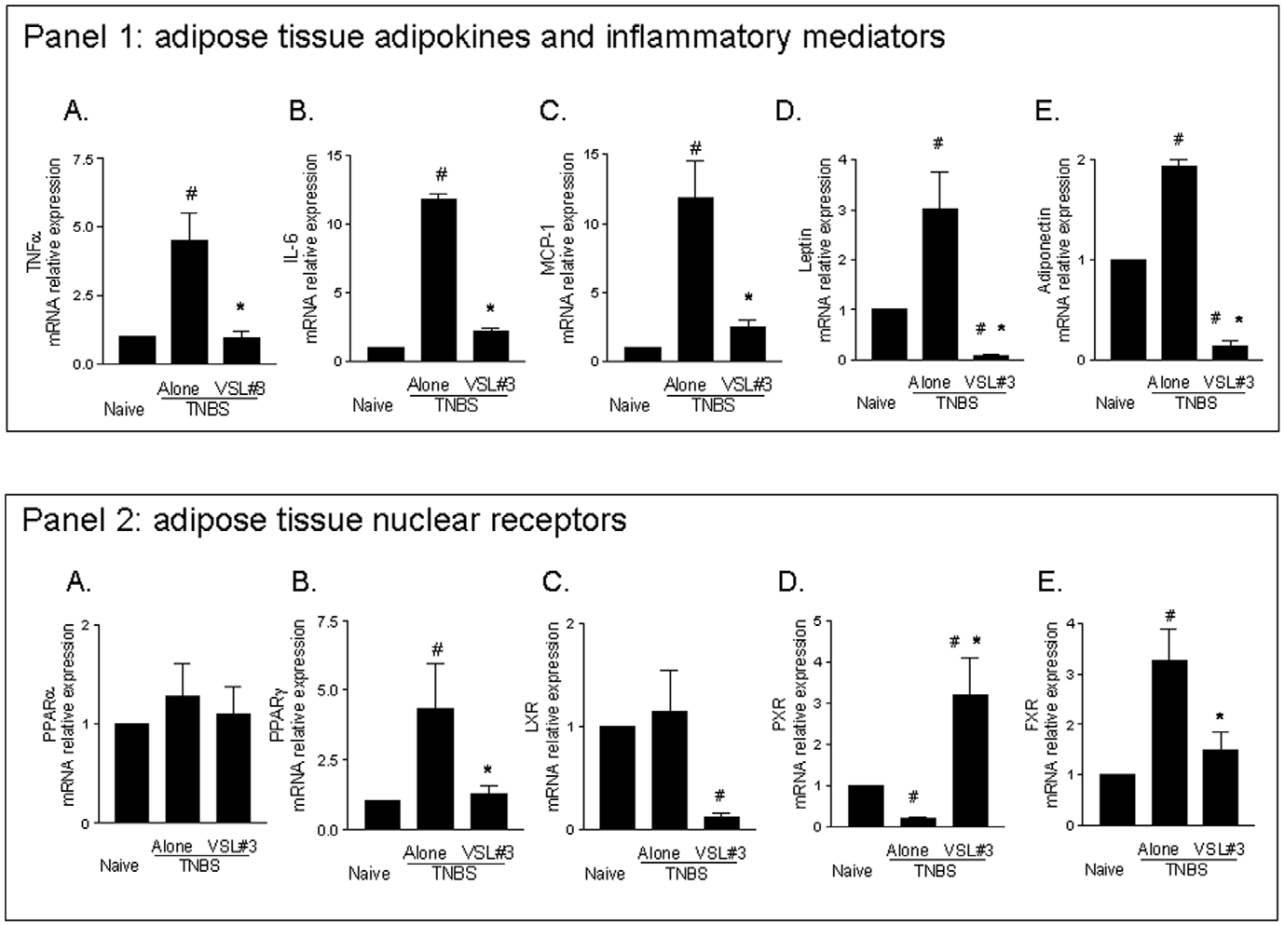
VSL#3 attenuates inflammation-driven metabolic dysfunction in the mesenteric adipose tissue. (**Panel 1** **A-E**) RT-PCR analysis of expression of inflammatory cytokines (TNFα, IL-6, and MCP1) and adipokines (leptin and adiponectin) in mesenteric adipose tissues. Data represent the mean ± SE of 5 mice per group. (#p<0.05 versus naïve; *p<0.05 versus TNBS). (**Panel 2** **A-E**) RT-PCR analysis of the expression of PPARα, PPARγ, LXR, PXR and FXR in mesenteric adipose tissues obtained 5 days after administration of TNBS alone or in combination with VSL#3. Data represent the mean ± SE of 5 mice per group. (#p<0.05 versus naïve; *p<0.05 versus TNBS).

TNBS administration resulted in robust inflammatory changes in the abdominal fat. Histophatlogy analysis of abdominal fat isolated from TNBS treated mice revealed extensive venular congestion, neutrophil margination and diapedesis and perivascular accumulation of neutrophils in the adipose tissue indicating that TNBS-induced inflammatory changes in the colonic mucosa are reflected in the surrounding fat (Figure 2, panel B,E,H). The mesenteric fat depots from the TNBS treated mice were characterized by an increased levels of the proinflammatory cytokines, including TNF-α, IL-6 and MCP-1 compared to naive mice (Figure 4, panel 1 A, B and C respectively n = 5; #p<0.05 versus naïve). In addition we observed an induction in the expression of leptin and adiponectin mRNAs (Figure 4 D and E respectively n = 5; #p<0.05 versus naïve) in mesenteric fat of colitic mice. These changes were abrogated by VSL#3 administration (Figure 4 and Figure 3; n = 5; #p<0.05 versus naïve; *p<0.05 versus TNBS).

In contrast to the colon, we found that in comparison with fat tissue from naive mice, the expression of PPARγ and FXR was increased in the adipose tissue of colitic mice while LXR and PPARα were unchanged and PXR was downregulated (Figure 4, Panel 2; n = 5; #p<0.05 versus naïve). VSL#3 coadministration restored the PPARγ and FXR mRNA levels (Figure 4, Panel 2; n = 5; *p<0.05 versus TNBS) while reduced drastically the LXR expression (Figure 4, Panel 2; n = 5; #p<0.05; *p<0.05 versus TNBS). The expression of PXR was upregulated (Figure 4, Panel 2; n = 5; #p<0.05 versus naïve; *p<0.05 versus TNBS).

Finally, we have investigated whether VSL#3 treatment could attenuate signs and symptoms of an established colitis. For this purpose animals were administered with VSL#3 (50^9^ cfu/day) starting one day after TNBS inoculation. As illustrated in Figure S3, this treatment attenuated colitis development as evaluated by assessing body weight changes, the macroscopic damage score and the MPO activity (n = 8-10. #p<0.05 versus naïve; *p<0.05 versus TNBS).

### Expression of nuclear receptors, adipokines in creeping fat and MAT explants from Crohn's disease patients

**Figure 5:**
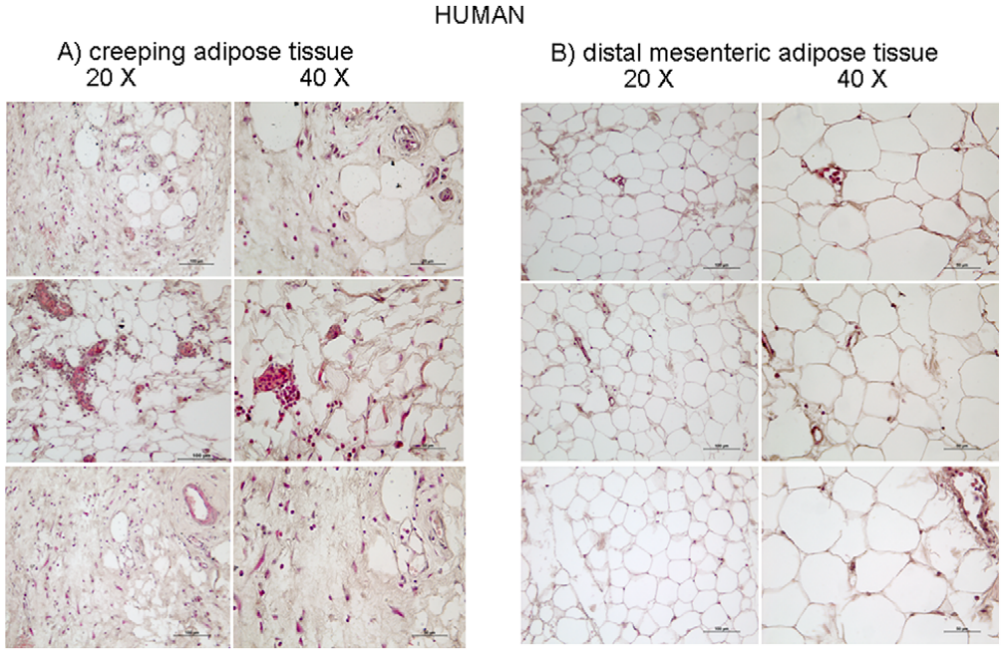
Distinctive histologic features the creeping fat and mesenteric adipose tissue in Crohn's disease patients. Rappresentative haematoxylin-eosin (H&E) staining of mesenteric adipose tissue of Crohn's patients. Creeping fat original magnification 10× and 20×, respectively (A) adipose tissue distal to intestinal mucosa of Crohn's disease patients, original magnification 20× (Bars: 100 µm) and 40×(Bars: 50 µm), respectively (B). Paired samples from three patients are shown.

**Figure 6:**
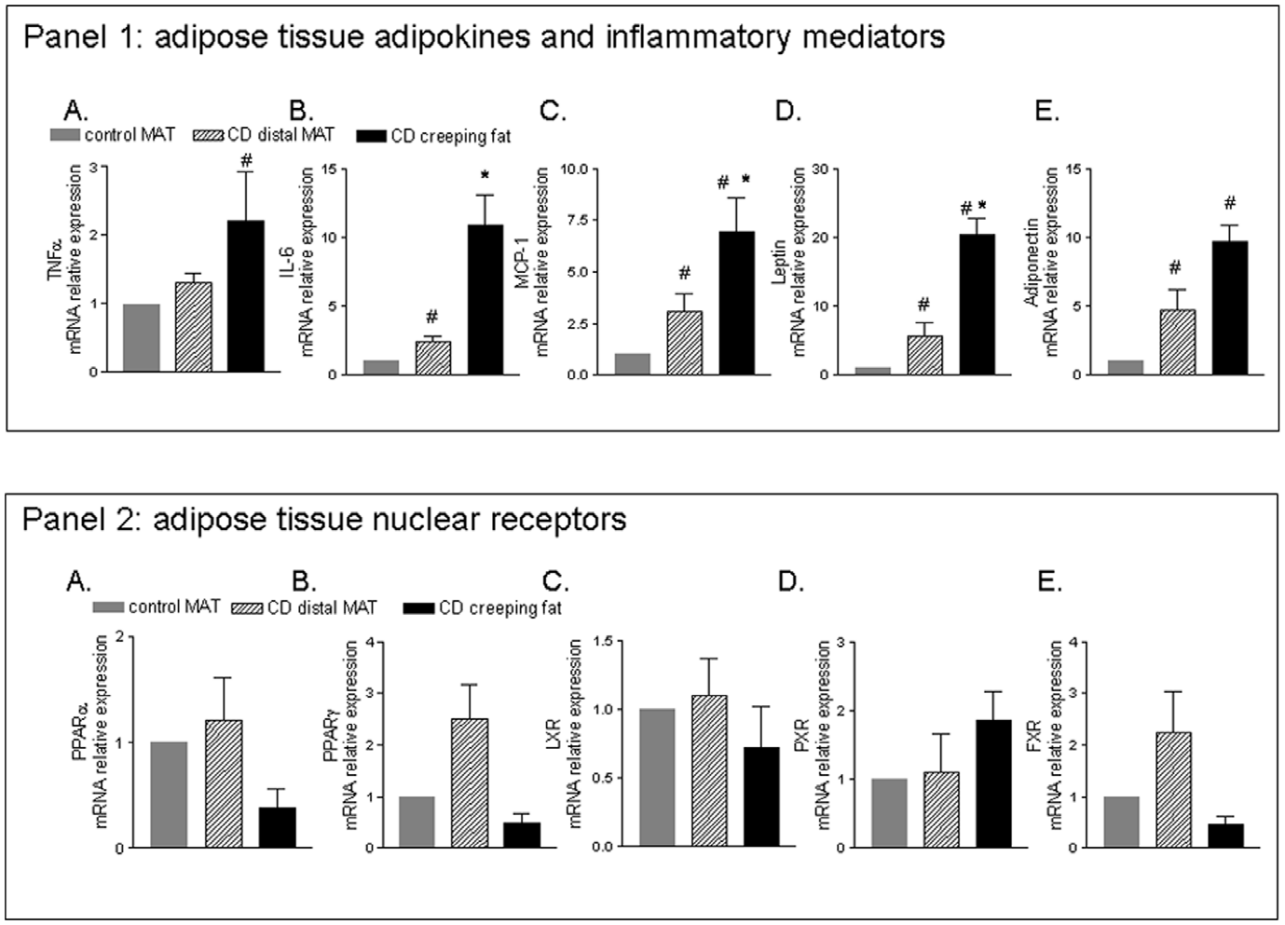
Expression of inflammatory mediators, adipokines and selected nuclear receptors in mesenteric adipose tissues from Crohn's patients. RT-PC analysis of expression of inflammatory TNFα, IL-6, MCP1, leptin and adiponectin **(Panel 1 A-E)** and nuclear receptors (PPARα, PPARγ, LXR, PXR and FXR) **(Panel 2 A-E)** in mesenteric adipose tissue (MAT) obtained from control subjects (N = 5) (colon carcinoma) and in Crohn's patients (creeping fat and distal MAT) (N = 5). Data represent the mean ± SE of 5 different subjects per group. (#p<0.05 versus MAT of control subjects ; *p<0.05 versus distal MAT obtained from Crohn's disease patients).

Histopathological examination of mesenteric fat explants form Crohn's disease patient demonstrate a large number of inflammatory cells in creeping adipose tissue (Figure 5 A). In contrast, very few inflammatory cells were detected in distal mesenteric adipose tissue (Figure 5 B). Consistent with these changes creeping fat explants were characterised by increased mRNA levels of the proinflammatory cytokines such as TNFα, IL-6, MCP-1 and leptin and adiponectin (Figure 6, panel 1 B, C, D and E respectively n = 5; * p<0.05). However, MAT explants obtained from Crohn's disease patients had a significant higher level of expression of inflammatory mediators (IL-6, MCP-1) and leptin and adiponectin when compared to MAT obtained from patients with colon carcinomas (Figure 6, panel 1; n = 5; # p<0.05). The creeping fat was also characterized by a decreased expression of PPAR α and γ and FXR compared to MAT from Crohn's disease patients and control subjects (Figure 6, panel 2).

**Figure 7:**
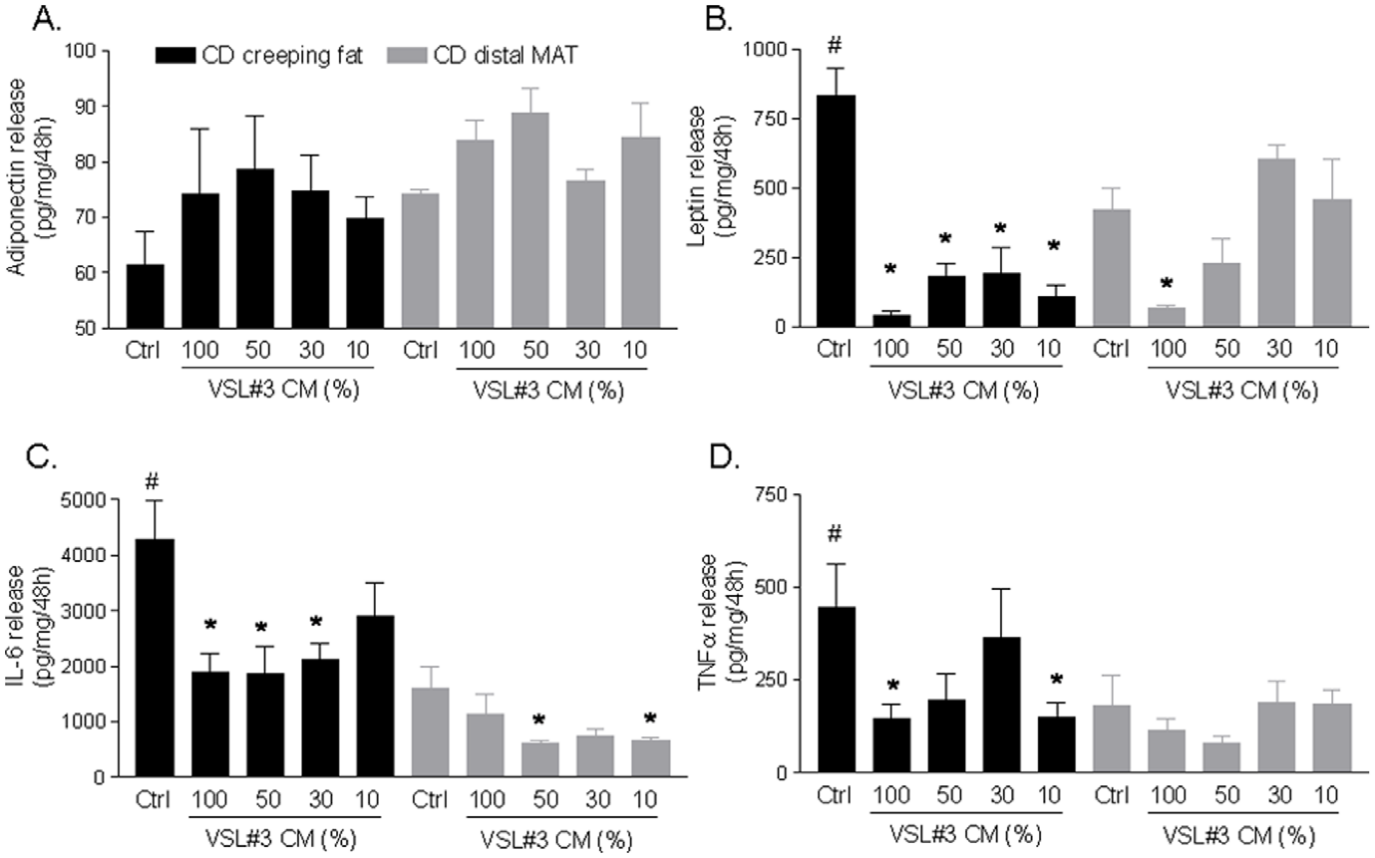
VSL#3 CM modulates the production of mesenteric adipose tissue factors. Release of adiponectin, leptin, IL-6 and TNFα by adipose tissue explants. Release by proximal (creeping fat) and distal MAT explants from 5 Crhon's patients is shown. Creeping fat and MAT explants were cultured alone or in combination with different concentrations of VSL#3 CM for 48 h. (#p<0.05 basal production (Ctrl) creeping versus MAT; n = 25; * p<0.05 verus control group, n = 25).

To determine whether VSL#3's bacteria secrete factors possessing anti-inflammatory activity, creeping fat and MAT explants from Crohn's disease were cultured with increasing concentrations of VSL#3 CM for 48h. A shown in Figure 7, explants from creeping fat released significant higher levels of leptin, IL-6 and TNFα compared to MAT (#p<0.05; n = 25). Interestingly, treatment with VSL#3-CM (all doses) reduced drastically the leptin production by creeping adipose tissue (Figure 7 B; *p<0.05; n = 25). In addition VSL#3-CM reduced the production of IL-6 and TNFα (Figure 7 C and D; *p<0.05; n = 25).

## Discussion

Probiotics which deliver some of the beneficial immunomodulatory effects of the commensal gut microbiota and induce immune homeostasis are an effective treatment for mild to moderate IBD [24]–[29]. Here we report that VSL#3 administration is effective in preventing development of an acute colitis induced by TNBS and found that protection correlates with a robust attenuation of inflammation as measured by assessing the colitis macroscopic score, neutrophil infiltration and the mRNA levels of TNFα, IL-1β, IL-6 and IFNγ.

Nuclear receptors are a superfamily of regulatory factors that exert homeostatic functions in the intestine at the interface between nutrients metabolism and immunity. Activation of PPARá and ã, PXR, FXR and LXR by lipid mediators, bile acids and oxysterols modulates lipid/cholesterol metabolism, provides counter-regulatory signals for macrophages and protection in rodent models of dysregulated innate immunity [18], [30]. A confirmation of the inverse correlation existing between nuclear receptors expression/activity and host susceptibility to inflammation in the intestinal compartment, comes from the observation that acute exposure to TNBS results in a downregulation of colonic expression of PPARã, PXR, and FXR. Of relevance, VSL#3 counter-reacts the effect of TNBS on inflammation and preserves the intestinal expression of these nuclear receptors.

The adipose tissue is an important source of hormones and cytokines [31]. Patients with Crohn's disease accumulate adipokine-releasing intra-abdominal fat from the onset of the disease [32], [33] indicating that expansion of mesenteric fat depots may be an important source of inflammatory mediators in IBDs [34]. Here we have shown that acute colitis induced by TNBS in mice associates with inflammatory changes in the mesenteric fat depots sampled proximally to the inflamed intestine. These changes are characterised by infiltration of mesenteric fat by leucocytes and increased expression of TNFα, IL–6, MCP-1, that have a mechanistic relevance in the development of systemic manifestations of IBDs [35].

The key finding of the present study, however, is that this inflammation-driven metabolic activation of adipose tissue is mediated by a differential regulation in the expression and several nuclear receptors. An abnormal expression of PPARγ, a nuclear receptor which is predominantly expressed in adipocytes and involved in adipogenesis, has been reported in the MAT of Crohn's patients [32]. Activation of PPARγ has important functionl consequences. Indeed PPARγ is a potent inducer of adipocytes growth and differentiation promoting their transition from small, quiescent, adipocytes to large, activated, adipocytes [36]–[38]. The fact that TNBS induced acute inflammation exerts different effects on PPARγ, with a reduced expression in the colon and an increased expression in the mesenteric fact, seems to support a role for this nuclear receptor in promoting the acquisition of a pro-inflammatory phenotype by mesenteric adipocytes. A similar pattern of expression was observed with FXR. Indeed, while colitis associates with a reduced expression of FXR in the colon, the expression of this nuclear receptor was robustly increased in the mesenteric fat of TNBS-treated mice. We and other have shown that FXR is involved in adipocytes differentiation, adipogenesis, and lipid storage in *vivo* and in *vitro* increasing the adiponectin through a mechanism that is partially mediated by PPARγ [39]–[40].

In contrast to mesenteric fat depots obtained from TNBS mice, we have observed that in comparison to MAT obtained from control subjects and Crohn's patients, creeping fat tissue obtained from Crohn's disease patients is characterized by an increased expression of IL-6, MCP-1 and leptin but has a reduced expression of PPARα, PPARγ and FXR. In contrast, in comparison to MAT from control subjects, MAT tissue from Crohn's patients was characterised by an increased expression of IL-6, MCP-1, leptin and adiponectin, as well as an increased expression of PPARγ and FXR. These changes seem to support a different biological roles of creeping fat and MAT in Crohn's disease. However, it is noteworthy that even MAT was characterized by the expression of a subset of genes known to support an active adipocytes differentiation, i.e.PPARγ and FXR [39], [40], [41]. In aggregate these data seems to suggest that activation of mesenteric adipose tissue is common in Crohn's patients and might contribute to development of local and systemic signs of disease.

IBDs, including Crohn's disease, are characterized by an increase in mucosal permeability allowing luminal molecules to travel through the intestinal wall or from lymphatic vessel to surrounding tissues. Thus we have investigated whether probiotics release factors that might drive the inflammatory response in the adipose tissue. Interestingly we found that, while creeping fat releases higher levels of leptin, IL-6 and TNFα compared to the MAT explants obtained from Crohn's disease patients, incubation with VSL#3-CM conter-regulates the production of these inflammatory mediators and abrogates the generation of leptin. Leptin is an adipocyte-secreted hormone that regulates the size of adipose tissue mass [42], reduces food intake and increases the metabolic rate [42]. Despite the correlation between body mass index and plasma leptin is usually preserved in IBDs [43], the mechanistic role of hyperleptinaemia in the onset of anorexia and weight loss associated with IBDs remains unclear [44]. However, it well recognized that circulating levels of leptin does not accurately reflect the local production of this hormone. Indeed a lack of correlation between leptin mRNA levels and plasma levels in IBDs has already been reported [45], reinforcing the notion that leptin produced by MAT might not contribute significantly to the plasma level of the hormone, but rather contributes to a local paracrine effect in the intestine and mesenteric fat. In fact, leptin increases TNFα secretion [45] and promotes changes in the balance between Th1 and Th2 cytokines increasing the generation of Th1 cytokines (IFN-γ and IL-2) and repressing IL-4 [45]. It is also well recognized, that in the other tissue, such as the liver, leptin promotes the activation of locally resident fibroblasts and leptin production associates with liver fibrosis [46]. Because activation of intestinal fibroblasts by locally released mediators and enhanced deposition of extracellular matrix is a prototypical features in Crohn's disease, is could be speculated that the uncheked leptin production we documented by creping fat and MAT might contribute to intestinal fibrosis in Crohn's patients. The demonstration that VSL#3-CM attenuates leptin production from MAT and creeping fat explants might have therefore a role in explaining the clinical effects of this agent, and paves the way to develop specific therapies.

The increasing awareness that mesenteric and creeping fat might contribute to inflammation and perhaps, to fibrosis, in Crohn's disease and that activity of these tissues could be regulated through a differential expression of nuclear receptors, raises the possibility to target these tissues with selective ligands for these transcription factors. Selective PPARγ agonists exerts anti-inflammatory effects while regulating major metabolic pathways in the abdominal fat and have been demonstrated useful in reducing intestinal inflammation in IBDs [47]. However, the use of PPARγ ligands associates with an increased risk of cardiovascular ischemic events, at least in the case of rosiglitazone [48]. Present results and previous data demonstrate that FXR exerts an anti-inflammatory activity in rodent models of colitis [18] while promoting a less activated phenotype in the adipose tissue [39], suggesting a potential therapeutic role for ligands of this nuclear receptor in the treatment of inflammation-driven activation of adipose tissue in Crohn's disease. However, further investigations are required to better define interactions between intestinal inflammation and mesenteric fat activation and whether mesenteric fat could be targeted by nuclear receptor modulators.

In conclusion, we have shown that colonic inflammation regulates the expression of several nuclear receptor in MAT in a model of colitis and in Crohn's disease patients. MAT activation could contribute to inflammation-driven immune and metabolic dysfunction in these patients by generating a subset of pro-inflammatory mediators and modulating the expression of several nuclear receptors. These effects are counter-regulated by changing the composition of enteric flora with a probiotic.

## Materials and Methods

### Animals

CD1 mice, 8 weeks of age, were obtained from Harlan Nossan (Udine, Italy). Mice were housed under controlled temperatures (22°C) and photoperiods (12∶12-hour light/dark cycle), allowed unrestricted access to standard mouse chow and tap water and allowed to acclimate to these conditions for at least 5 days before inclusion in an experiment. Protocols were approved by the University of Perugia Animal Care Committee according to the Italian guideline for care and use of laboratory animals. The ID for this project is #98/2010-B. The authorization was released to Prof. Stefano Fiorucci, as a principal investigator, on May 19, 2010.

### Reagents

Purified myeloperoxidase (MPO) and tri-methylbenzidine, trinitro-benzene sulfonic acid (TNBS) were obtained from Sigma-Aldrich (Milan, Italy). The probiotics compound VSL3, consisting of 8 strains of bacteria (*L. acidophilus* MB 443, *L. delbrueckii* subsp. *bulgaricus* MB 453, *L. casei* MB 451, *L. plantarum* MB 452, *B. longum* Y10, *B. infantis* Y1, *B. breve* Y8, and *S. salivarius* subsp. *thermophilus* MB 455), was obtained from VSL Pharmaceuticals.

### Experimental Procedures

Mice were lightly anesthetized by intraperitoneal injection of 100 µl of ketamine/xylazine solution [18], [21] per 10 g body weight and then administered intrarectally (i.r.) with the haptenating agent TNBS (1.5 mg/mouse) dissolved in ethanol 50%, via a 3.5 French (F) catheter equipped with a 1-ml syringe. The catheter was advanced into the rectum for 4 cm and then the haptenating agent was administered in a total volume of 150 µl. To ensure distribution of the agent within the entire colon and cecum, mice were held in a vertical position for 30 seconds. An additional control group was obtained by administering mice with ethanol alone on day 6. Animals (n = 5) were followed for body weight changes for 7 days.

Treated animals received, orally, saline or probiotics, daily, at dose of 50×10^9^ colony-forming units (cfu)/kg/day (1.25×10^9^/mouse) (n = 10–8 for each group), 5 days before induction of colitis. Mice were sacrificed 5 days after TNBS administration. The mice were monitored daily for weigh loss and fecal score. Probitics were dissolved each day in physiologic solution and administrated orally at the final volume of 200 µL/mouse. The TNBS group mice received the vehicle alone every day. Five days after TNBS administration, surviving mice were sacrificed, colons and mesenteric adipose tissue were removed and immediately snap-frozen in liquid nitrogen and stored at −80°C until use. The macroscopic appearance was analyzed under a dissecting microscope (× 5) and graded for macroscopic lesions on a scale from 0 to 10 based on criteria reflecting inflammation, such as hyperemia, thickening of the bowel, and the extent of ulceration. Neutrophil infiltration in the colon was monitored by measuring MPO activity using a spectrophotometric assay with tri-methylbenzidine (TMB) as a substrate. Activity is expressed as mU per mg protein.

### Patients

MAT and creeping fat explants were obtained from five patients affected by Crohn's disease (2 women; mean age 36±8 years old). All patients underwent right ileocolonic resection because of symptomatic ileal stenosis with transmural inflammation. All patients had a stenotic disease complicated buy by ileocolonic abscesses in 3 patients and an intestinal fistula, 2 patients. All were treated with antibiotic (ciprofloxacin and metronidazole) for before surgery. Anti-TNFα therapy was the main therapy in two patients. All had been treated with azathioprine in the weeks before the surgery. Five subjects with carcinoma of the right colon (2 women ; mean age 47±8 years old) served as controls. None of these control subjects was obese. In patients with colon carcinoma mesenteric fat samples were obtained in front of normal intestine at a sufficient minimal distance from tumour. Adipose tissue samples were immediately frozen in liquid nitrogen and stored at −80°C for subsequent mRNA analysis or processed to tissue culture biopsy. An informed written consent on the use of removed surgical samples was obtained by each patient. The authorization of an ethical committee was not requested because for small exploratory studies, by internal hospital guidelines, only the informed consent by the patient is requested.

### VSL#3 conditioned media preparations and human adipose tissue cultures

To prepare conditioned medium (CM), 10 mg of VSL#3 probiotics formula was reconstituted in 10 ml of serum/antibiotic-free Dulbeccos Modified Eagle's cell culture medium and was grown overnight in medium at 37°C without shaking. The CM was centrifuged at 4,100 rpm for 10 min to separate the bacteria, and the resulting supernatant was filtered two times through a 0.22- µm membrane (Millipore) to remove any insoluble particles and diluted with DMEM cell culture medium free of serum and supplemented with to have a final concentration of 10 mg/ml bovine serum albumin, 5 µg/ml ethanolamine, 0.1 ng/ml sodium selenite, 100 U/mL penicillin and 100 µg/mL streptomycin, 50 µg/ml gentamicin and 55 µM ascorbic acid. The pH of the buffer was adjusted to 7.4 and then filtered through a 0.22- µm filter (100-50-30-10%). The fat tissue samples were surgically removed and placed in Hanks' Balanced Salt Solution, (HBSS) supplemented with 100 U/mL penicillin and 100 µg/mL streptomycin. Fat tissue lobules were prepared under sterile conditions by microdissection. Vessels and adjacent soft tissue were carefully removed. After preparation, the fat tissue samples were washed four time with HBSS supplemented with 300 U/mL penicillin and 300 µg/mL streptomycin. At least 25 fat tissue specimens, creeping and distal, were cultured from each patient and secretion of Leptin, Adiponectin, IL-6 (Orgenium Laboratories) and TNFα (SABioscence) was determined. Based on this procedure, a total number of n = 25 tissue samples was incubated from 5 CD patients, alone or in combination VSL#3 CM (100-50-30-10%). The tissue samples were incubated in 1 mL medium for 48 h at 37°C in a 95% O2 and 5% CO2 incubator. Supernatants were collected and stored at−20°C. The wet weight of fat tissue was measured (60–80 mg) in order to express the cytokine secretion as pg/mg fat per 48 h.

### Histological Analysis

For histological examination, tissues were fixed in 10% buffered formalin phosphate, embedded in paraffin, sectioned, and stained with hematoxylin and eosin (H&E). Histology images were captured by a digital camera (Digital Microscope Camera ProgResC14, Jenoptik, Germany) and analyzed by specific software (Delta Sistemi, Rome, Italy).

### Real-Time PCR

Quantization of the expression level of selected genes was performed by quantitative real-time PCR (qRT-PCR). Total RNA were obtained from colon and adipose tissue pieces (100–50 mg) and isolated with TRIzol reagent (Invitrogen, Milan, Italy), incubated with DNase I and reverse-transcribed with Superscript II (Invitrogen) according to manufacturer specifications. For real-time PCR, 50–25 ng of template was used in a 25- µl reaction containing a 0.3 µM concentration of each primer and 12.5 µl of 2x SYBR Green PCR Master Mix (Bio-Rad Laboratories, Hercules, CA). All reactions were performed in triplicate using the following cycling conditions: 2 min at 95°C, followed by 50 cycles of 95°C for 10 s and 60°C for 30 s using an iCycler iQ instrument (Bio-Rad Laboratories). The mean value of the replicates for each sample was calculated and expressed as cycle threshold (C_T_). The amount of gene expression was then calculated as the difference (ΔC_T_) between the C_T_ value of the sample for the target gene and the mean C_T_ value of that sample for the endogenous control (GAPDH). Relative expression was calculated as the difference (ΔΔC_T_) between the ΔC_T_ values of the test and control samples for each target gene. The relative level of expression was measured as 2^−ΔΔCT^. All PCR primers were designed using the software PRIMER3-OUTPUT using published sequence data obtained from the NCBI database.

Mouse and Human primers were as follows:

mGAPDH: CTGAGTATGTCGTGGAGTCTAC and GTTGGTGGTGCAGGATGCATTG;

mIL1β: TCACAGCAGCACATCAACAA and TGTCCTCATCCTCGAAGGTC;

mIL-6: CCGGAGAGGAGACTTCACAG and TCCACGATTTCCCAGAGAAC;

mIL-10: GCTGGACAACATACTGCTAACC and CTGGGGCATCACTTCTACCA;

mINFγ: GCGTCATTGAATCACACCTG and GACCTGTGGGTTGTTGACTC;

mMCP-1: CCCAATGAGTAGGCTGGAGA and TCTGGACCCATTCCTTCTTG;

mTNFα: ACGGCATGGATCTCAAAGAC and GTGGGTGAGGAGCACGTAGT;

mTGFβ: TGGCTTCAGCTCCACAGAGA and TGGTTGTAGAGGGCAAGGAC;

mLeptin: TTCACACACGCAGTCGGTAT and TCATTGGCTATCTGCAGCAC;

mAdiponectin: ACAATGGCACACCAGGCCGT and CCCTTAGGACCAAGAAGACCTGCA;

mPPARα: CAGAGGTCCGATTCTTCCAC and GATCAGCATCCCGTCTTTGT;

mPPARγ: GCCAGTTTCGATCCGTAGAA and AATCCTTGGCCCTCTGAGAT;

mLXR: GCAGGACCAGCTCCAAGTAG and GGCTCACCAGCTTCATTAGC;

mPXR: ACGGCAGCATCTGGAACTAC and TGGTCCTCAATAGGCAGGTC;

mFXR: TGTGAGGGCTGCAAAGGTTT and ACATCCCCATCTCTCTGCAC.

hPPARa: ACGATTCGACTCAAGCTGGT and GTTGTGTGACATCCCGACAG;

hPPARg: GCTGGCCTCCTTGATGAATA and TTGGGCTCCATAAAGTCACC;

hPXR: AGCTGGAACCATGCTGACTT and CACATACACGGCAGATTTGG;

hFXR: TACATGCGAAGAAAGTGTCAAGA and ACTGTCTTCATTCACGGTCTGAT;

hTNFa: AACCTCCTCTCTGCCATCAA and GGAAGACCCCTCCCAGATAG;

hIL6: AGGAGACTTGCCTGGTGAAA and CAGGGGTGGTTATTGCATCT;

hMCP1: CCCCAGTCACCTGCTGTTAT and TCCTGAACCCACTTCTGCTT;

HLXR: CGCACTACATCTGCCACAGT and TCAGGCGGATCTGTTCTTCT;

hLeptin: GGCTTTGGCCCTATCTTTTC and GCCAGTTCTGGTCCATCTT;

hAdiponectin: CCTGGTGAGAAGGGTGAGAA and GTAAAGCGAATGGGCATGTT.

### Statistical analysis

All values are expressed as the mean ± SE of n mice per group. Comparisons of more than 2 groups were made with a one-way analysis of variance with post hoc Tukey tests. Differences were considered statistically significant if p was <0.05.

## Supporting Information

Figure S1 **LPMC were isolated from freshly obtained colonic specimens.** After excision of all visible lymphoid follicles, colons were digested with type IV collagenase (Sigma) for 20 min in a shaking incubator at 37°C; this step was repeated twice. The released cells were then layered on a 40%-100% Percoll gradient (Pharmacia, Upsala, Sweden) and spun at 1,800 rpm to obtain the lymphocyte-enriched populations at the 40–100% interface. For flow cytometry analysis 0.8×10^6^ LPMC obtained from naïve and TNBS (1.5 mg/mouse) treated mice (4 after colitis induction) alone or in combination with VSL#3 (50×109 colony-forming units (cfu)/kg/day) for 5 days before induction of colitis. Cells were stained (20 min at 4°), with specific mAbs against CD3, CD14, CD19, and NK-1.1 (phycoerythrin (PE) or fluorescein isothiocyanate (FITC)--conjugated) (BD Biosciences). At the end of incubation, cells were washed two times with phosphate buffered saline (PBS) buffer and resuspended in PBS containing formaldehyde (4%) prior to flow cytometric analysis (Epics XL-2; Beckman Coulter, USA).
(PPT)

Figure S2 **Total lysates from colon were prepared by E1A-buffer.** Protein levels in tissue extract were quantified with Bradford reagent. Proteins, 30 µgrams, (a pool of 5 different animals, 6 µgrams each) were separated by polyacrylamide gel electrophoresis, transferred to nitrocellulose membranes (Bio-Rad, Hercules, CA) and than probed with primary anti-FXR antibody (0.5 µg/ml) (Ab 28676, Abcam). The anti-immunoglobulin G Rabbit (Bio-Rad) was used as a secondary antibody, and specific protein bands were visualized by chemoluminescence using Supersignal West Dura reagent (Pierce, Rockford, IL).
(PPT)

Figure S3 **Colitis was induced in Balb/c by intrarectal administration of TNBS (0.5 mg/mouse) in 50% ethanol.** To assess whether administration of VSL#3 would protect against development of colitis, TNBS-treated mice were randomized to receive vehicle or probiotics, daily, (the day after TNBS administration) at dose of 50×10^9^ colony-forming units (cfu) (n = 10 for each group). The mice were monitored daily for weigh loss and fecal score (A and B). The macroscopic appearance was analyzed under a dissecting microscope (x 5) and graded for macroscopic lesions on a scale from 0 to 10 based on criteria reflecting inflammation, such as hyperemia, thickening of the bowel, and the extent of ulceration (C). Neutrophil infiltration in the colon was monitored by measuring MPO activity using a spectrophotometric assay with tri-methylbenzidine (TMB) as a substrate (D). Activity is expressed as mU per mg protein.*P value <.05 was considered significant vs Naive group. *#P* value <.05 was considered significant vs TNBS group. The ANOVA test was used for statistical comparisons.
(PPT)
