# Supplementary figures and images for: Probiotics Modulate Intestinal Expression of Nuclear Receptor and Provide Counter-Regulatory Signals to Inflammation-Driven Adipose Tissue Activation
Source: PLoS One. 2011 Jul 29;6(7):e22978. doi: 10.1371/journal.pone.0022978 (PMC3146529; doi:10.1371/journal.pone.0022978)

## Slide 1
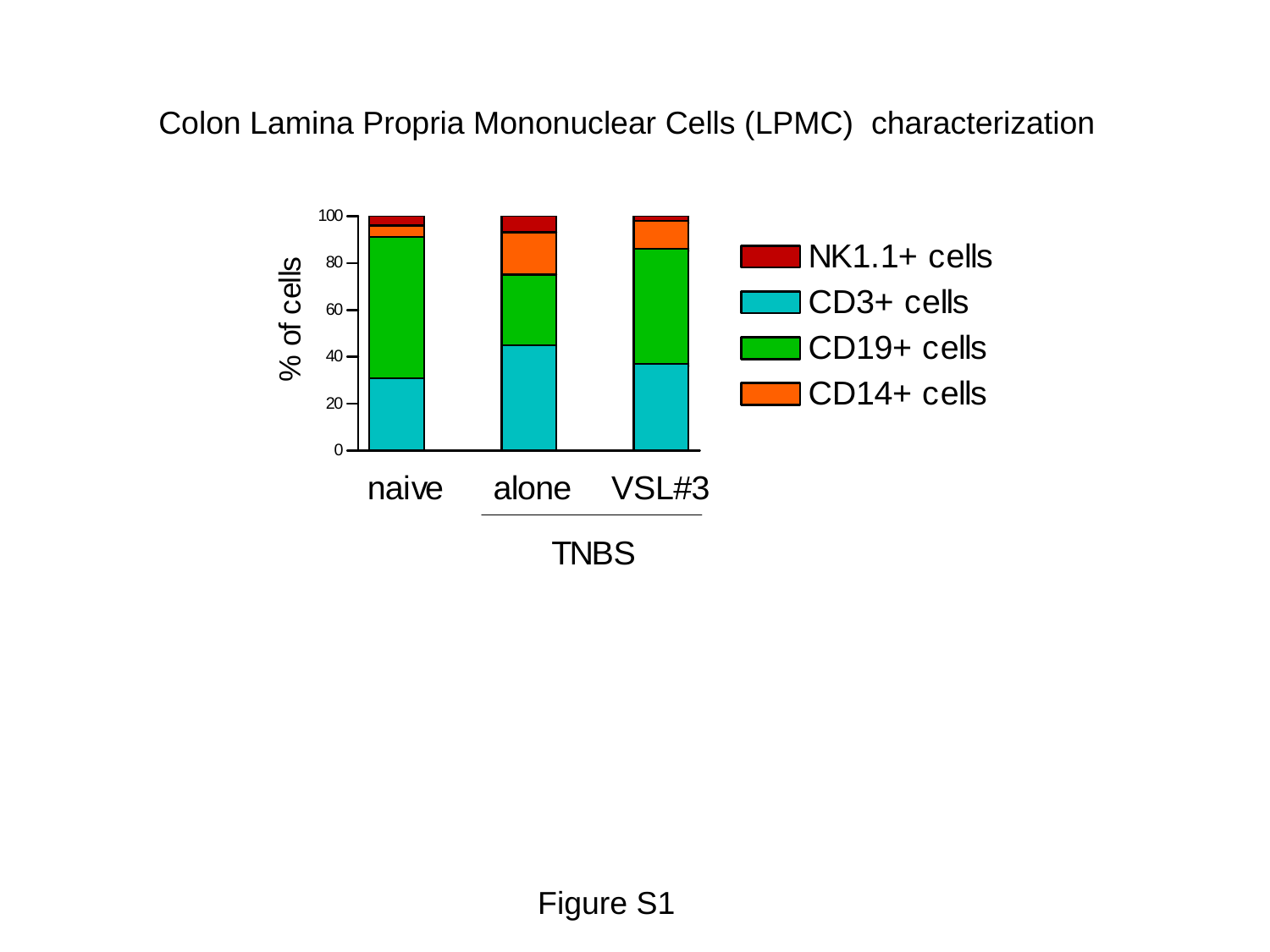

Colon Lamina Propria Mononuclear Cells (LPMC) characterization
Figure S1

Supplement: Figure S1 — LPMC were isolated from freshly obtained colonic specimens. After excision of all visible lymphoid follicles, colons were digested with type IV collagenase (Sigma) for 20 min in a shaking incubator at 37°C; this step was repeated twice. The released cells were then layered on a 40%-100% Percoll gradient (Pharmacia, Upsala, Sweden) and spun at 1,800 rpm to obtain the lymphocyte-enriched populations at the 40–100% interface. For flow cytometry analysis 0.8×10^6^ LPMC obtained from naïve and TNBS (1.5 mg/mouse) treated mice (4 after colitis induction) alone or in combination with VSL#3 (50×109 colony-forming units (cfu)/kg/day) for 5 days before induction of colitis. Cells were stained (20 min at 4°), with specific mAbs against CD3, CD14, CD19, and NK-1.1 (phycoerythrin (PE) or fluorescein isothiocyanate (FITC)--conjugated) (BD Biosciences). At the end of incubation, cells were washed two times with phosphate buffered saline (PBS) buffer and resuspended in PBS containing formaldehyde (4%) prior to flow cytometric analysis (Epics XL-2; Beckman Coulter, USA). (PPT) [file pone.0022978.s001.ppt]

## Slide 1
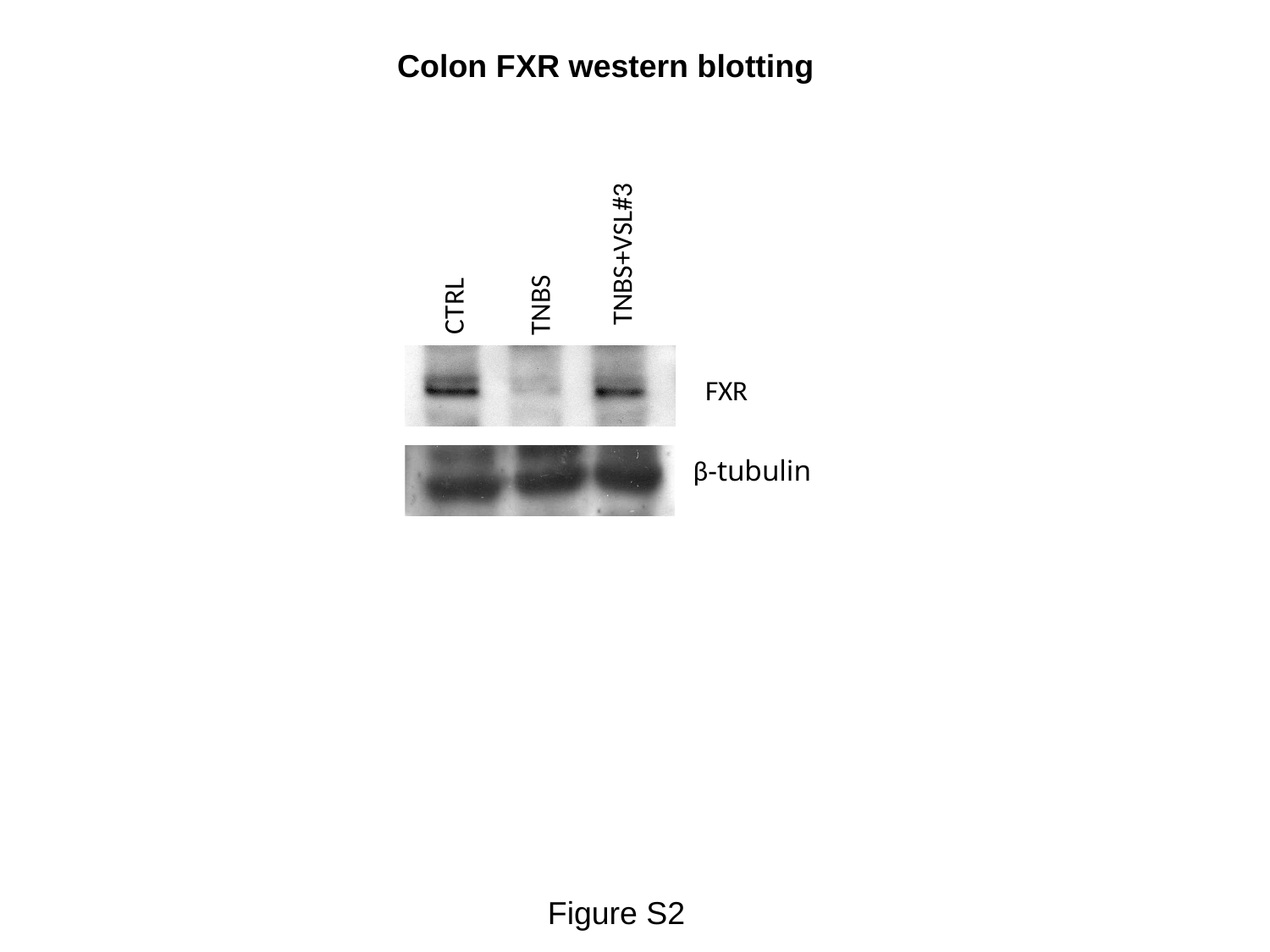

Colon FXR western blotting
TNBS+VSL#3
TNBS
CTRL
FXR
β-tubulin
Figure S2

Supplement: Figure S2 — Total lysates from colon were prepared by E1A-buffer. Protein levels in tissue extract were quantified with Bradford reagent. Proteins, 30 µgrams, (a pool of 5 different animals, 6 µgrams each) were separated by polyacrylamide gel electrophoresis, transferred to nitrocellulose membranes (Bio-Rad, Hercules, CA) and than probed with primary anti-FXR antibody (0.5 µg/ml) (Ab 28676, Abcam). The anti-immunoglobulin G Rabbit (Bio-Rad) was used as a secondary antibody, and specific protein bands were visualized by chemoluminescence using Supersignal West Dura reagent (Pierce, Rockford, IL). (PPT) [file pone.0022978.s002.ppt]

## Slide 1
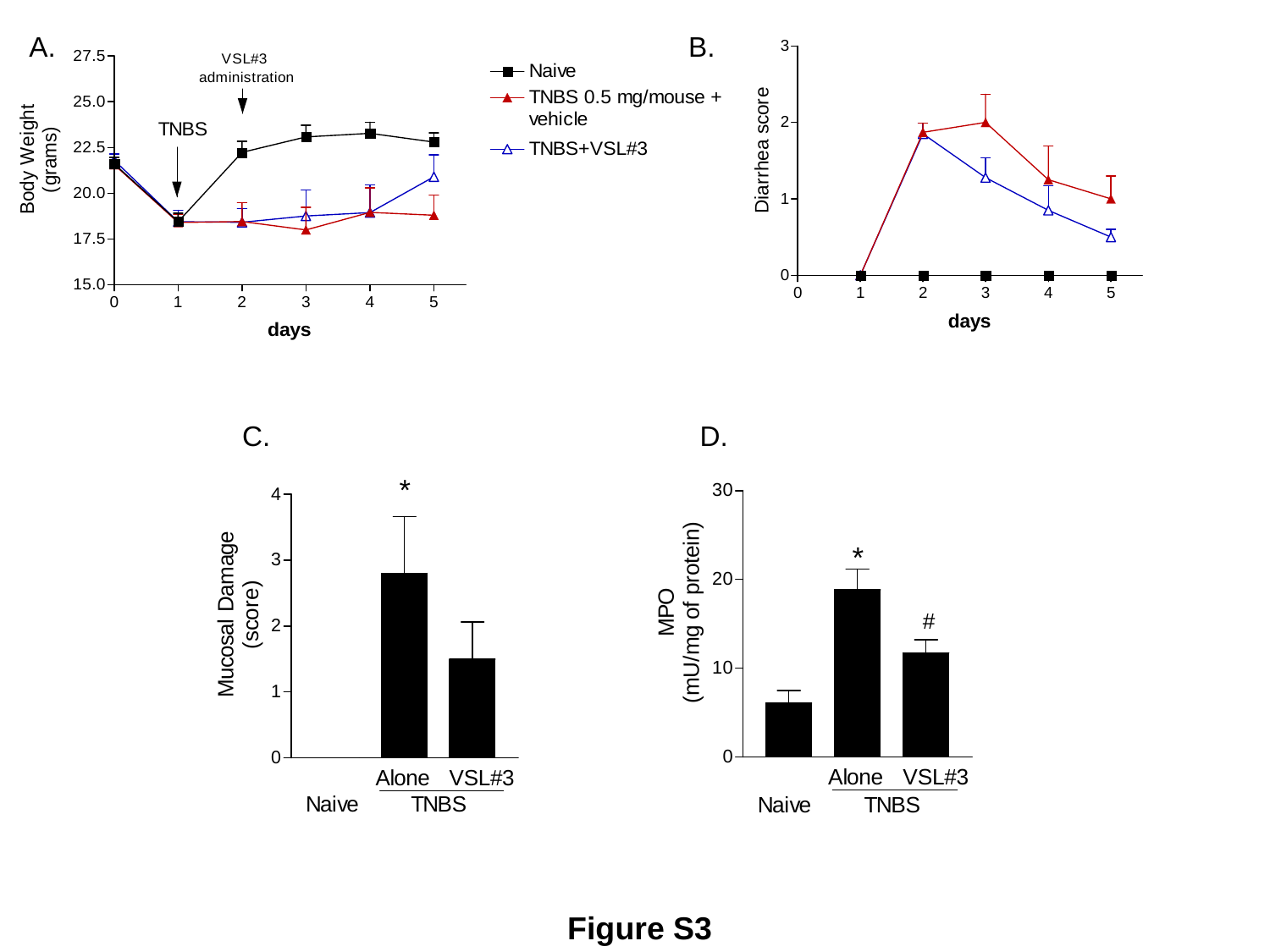

A. B.
C. D.
Figure S3

Supplement: Figure S3 — Colitis was induced in Balb/c by intrarectal administration of TNBS (0.5 mg/mouse) in 50% ethanol. To assess whether administration of VSL#3 would protect against development of colitis, TNBS-treated mice were randomized to receive vehicle or probiotics, daily, (the day after TNBS administration) at dose of 50×109 colony-forming units (cfu) (n = 10 for each group). The mice were monitored daily for weigh loss and fecal score (A and B). The macroscopic appearance was analyzed under a dissecting microscope (x 5) and graded for macroscopic lesions on a scale from 0 to 10 based on criteria reflecting inflammation, such as hyperemia, thickening of the bowel, and the extent of ulceration (C). Neutrophil infiltration in the colon was monitored by measuring MPO activity using a spectrophotometric assay with tri-methylbenzidine (TMB) as a substrate (D). Activity is expressed as mU per mg protein.*P value <.05 was considered significant vs Naive group. #P value <.05 was considered significant vs TNBS group. The ANOVA test was used for statistical comparisons. (PPT) [file pone.0022978.s003.ppt]
